# Fabrication and Characterization of a Low-Cost Microfluidic System for the Manufacture of Alginate–Lacasse Microcapsules

**DOI:** 10.3390/polym12051158

**Published:** 2020-05-19

**Authors:** Ana Lucia Campaña, Diana Camila Sotelo, Hector Alfonso Oliva, Andres Aranguren, Nancy Ornelas-Soto, Juan C. Cruz, Johann F. Osma

**Affiliations:** 1 Department of Electrical and Electronic Engineering, Universidad de los Andes, Cra. 1E No. 19a-40, Bogotá D.C. 111711, Colombia; al.campana10@uniandes.edu.co (A.L.C.); dc.sotelo10@uniandes.edu.co (D.C.S.); ha.oliva10@uniandes.edu.co (H.A.O.); a.aranguren@uniandes.edu.co (A.A.); 2 Laboratorio de Nanotecnología Ambiental, Escuela de Ingeniería y Ciencias, Tecnológico de Monterrey, Monterrey 64849, Mexico; ornel@tec.mx; 3 Department of Biomedical Engineering, Universidad de Los Andes, Cra. 1E No. 19a-40, Bogotá D.C. 111711, Colombia; jc.cruz@uniandes.edu.co

**Keywords:** microfluidics, microcapsules, alginate, laccase

## Abstract

The development of microfluidics-based systems in the recent years has provided a rapid and controlled method for the generation of monodisperse microencapsulates for multiple applications. Here, we explore the design, manufacture and characterization of a low-cost microsystem for the encapsulation of the fungal laccase from *Pycnoporus sanguineus* CS43 in alginate microcapsules. Multiphysics simulations were used to overview the fluid behavior within the device and estimate the resulting capsule size. Polymethylmethacrylate (PMMA) sheets were used for final microsystem manufacture. Different flow rates of the continuous (Q_c_) and discrete (Q_d_) phases in the ranges of 83–293 mL/h and 1–5 mL/h, respectively, were evaluated for microcapsule fabrication. Universal Serial Bus (USB) microscope and image analysis was used to measure the final particle size. Laccase encapsulation was evaluated using spectrophotometry and with the aid of fluorescent dyes and confocal microscopy. Results showed microcapsule size was in the range of 203.13–716.00 μm and Q_c_ was found as the dominant parameter to control capsule size. There was an effective enzyme encapsulation of 65.94% with respect to the initial laccase solution.

## 1. Introduction

Microencapsulation technology has proved itself useful in a large variety of applications such as cosmetics, the protection and preservation of aroma or taste in the food industry or even pharmacological components that are sensitive to certain environmental or physiological conditions [1,2,3,4]. In this regard, perhaps one of the most attractive commercial applications is the development of multiple encapsulated products for various industries including agriculture [5], pharmacy [6], medicine [7,8], cosmetics and food [3,6]. This stems from the fact that microencapsulation provides an efficient route to protect or isolate substances from harmful environments, in which the compounds can be chemically unstable and susceptible to oxidative degradation [6]. Additionally, the encapsulated substances can be released from the entrapment material in a controlled manner, which is especially useful for selective delivery of medications. Moreover, this process occurs without significantly altering the properties of the core materials, thereby maintaining their specific functionalities.

Multiple manufacturing methods have been implemented in recent years to encapsulate an ample variety of molecules including enzymes, essential oils, dyes, flavors, sweeteners and microorganisms. Encapsulation takes place using both physical and chemical methods, including air suspension [9], interfacial polymerization [1,10,11,12], solvent evaporation [13,14,15], coacervation [14,16], water-in-oil emulsion [17] and ionic gelation [17,18,19]. The preferred materials for encapsulation are both natural and synthetic polymers such as gelatin, cellulose, alginate, chitosan, Arabic gum, maltodextrin, polyamide and polyester [8,14,20,21,22,23,24]. This is due to their relatively high inertness, tractability, biodegradability, solubility, surface activity, low cost and also an important antioxidation effect. One of the most widely used polymers for microencapsulation is alginate, which is a polysaccharide extracted from brown seaweed that exhibits good biodegradability, high biocompatibility and low toxicity [25,26]. Alginate suffers mild gelation when in contact with divalent ions such as Ca^+2^, Mg^+2^ and Cu^+2^ by forming a stable polymer network [25,27], which is then useful for the production of stable complex microcapsules.

Some of the major challenges of conventional microcapsule manufacture methods include the precise control of morphology, size distribution, porosity and composition of the prepared materials [10,28]. As a result, the scaling-up of such processes has been considerably limited. Alternatively, recent studies have suggested that droplet-based microfluidic systems can be successfully used to produce monodisperse microcapsules [29,30,31]. This has been attributed to a more homogeneous reaction environment, the possibility of adding reagents at precise time and space intervals and the chance to reduce the amount of reagents required for manufacture [32]. In a droplet-based flow within a microfluidic device, highly monodispersed microcapsules are generated when two immiscible flow phases are put in contact. The size and dispersion of the microcapsules depend on several operating parameters such as channel geometries and flow rates [31]. The combination of parameters to obtain the desired attributes is usually attained experimentally by trial and error procedures [32,33].

One of the main drawbacks of microfluidic devices is the need for sophisticated cleanroom techniques such as photolithography, spin-coating and thermal evaporation in a sterile environment. This has considerably limited the application of microfluidics at the industrial scale [30,34,35]. Thus far, some attempts to overcome this major hurdle include parallelization of microfluidic systems for droplet formation [36], as well as soft lithography techniques [37]. We have recently developed an alternative approach for the low-cost manufacturing of such devices that involves the use of laser-cutting techniques and the use of commercially available and inexpensive hose connectors. This approach has allowed us to conduct inexpensive rapid prototyping and proof-of-concept experiments.

Here, we aimed at preparing monodisperse alginate-based microcapsules using a low-cost microfluidic system and copper sulfate as cross-linker for the encapsulation of laccase enzymes. Copper was selected as cross-linking agent due to sodium alginate’s high selectivity for Cu^2+^ ions, the known antibacterial activity, the strong mechanical properties and its possible involvement in multiple biochemical reactions. Moreover, it has been classified as an essential nutrient, which make it useful for applications in smart wound dressings, agriculture and wastewater treatments [38,39,40,41]. The system was designed, simulated, manufactured and tested under different operation regimes to evaluate their impact on microcapsule size distribution and morphology as well as the encapsulation efficiency.

## 2. Materials and Methods

### 2.1. Materials

Copper (II) sulfate (CuSO_4_) (99%), N-(3-Dimethylaminopropyl)-N′-ethylcarbodiimide hydrochloride (EDC) (97%), N-Hydroxysuccinimide (NHS) (98%), Rhodamine B (RhB) (95%) and 2,2′-Azino-bis(3-ethylbenzothiazoline-6-sulfonic acid) diammonium salt (ABTS) (98%) were purchased from Sigma-Aldrich (St. Louis, MO, USA). Alginic Acid Sodium Salt BioChemica (Sodium alginate) (90.8%) was obtained from PanReac AppliChem (Barcelona, Spain). Commercial soybean vegetable oil, solvent-based acrylic glue and methylene chloride and polymethylmethacrylate (PMMA) sheets of 2 mm thickness were obtained from local distributors (Bogotá, Colombia).

Laccases (*P. sanguineus* CS43) (EC 1.10.3.2) were obtained from tomato medium as described elsewhere [42]. Briefly, mycelia were removed from the culture supernatant using filtration with two tangential flow filters in series, with pore sizes of 0.5 mm and 0.2 mm, respectively. The obtained laccase cocktail was ultra-filtered using a membrane with a cut-off of 10 kDa.

### 2.2. Microsystem Design and Multiphysics Simulation

**Figure 1:**
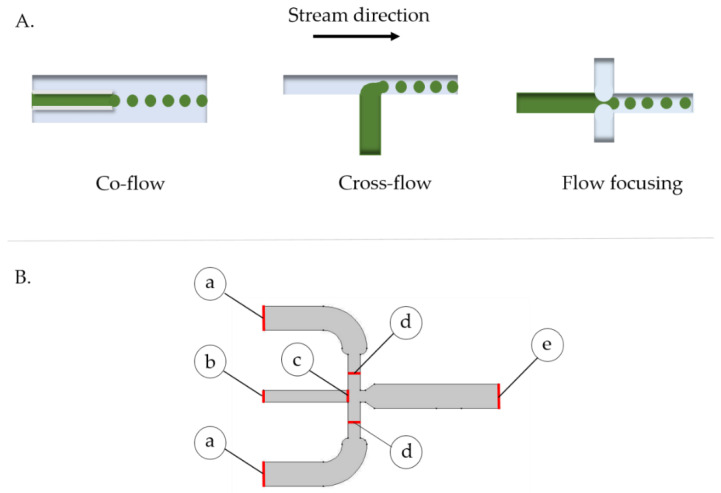
(**A**) Schematic of microfluidic system arrangements for microcapsule generation. Continuous phase is shown in blue while the discrete phase in green. (**B**) 2D geometry of the droplet generation junction in the designed microfluidic system. (**a**) Continuous phase inlet of 2 mm, (**b**) dispersed phase inlet of 1 mm, (**c**) initial interface of 1 mm, (**d**) continuous phase inlet of 1 mm and (**e**) flow outlet of 2 mm. Boundary conditions: no-slip wall condition$\;V_{h}=0$ mm/s, and pressure at the outlet $P_{o}=1\;\mathrm{atm},$ suppressing backflow.

The microsystem layout was selected from widely used designs for droplet generation from two phase interaction. The analyzed devices included those where the continuous (Q_c)_ and discrete (Q_d_) streams come into contact in co-flow, cross-flow and flow-focusing arrangements (Figure 1) [32]. Due to the possibility of having greater control over the size of the microcapsules by simply adjusting to the last section of the channel width, a flow-focusing approach was selected to manufacture the polymeric capsules.

The microsystem geometry was designed with the aid of the software Autodesk^®^ AutoCAD^®^ (Autodesk, Inc., San Rafae, CA, USA), according to the specifications shown in Figure 1B. COMSOL Multiphysics^®^ 5.3 software (COMSOL Inc., Stockholm, Sweden) was used to study momentum transfer and capsule generation within the device. This was accomplished by implementing the computational fluid dynamics (CFD) module of laminar two-phase flow, level set, which was analyzed via a temporal study. The level set approach allowed us to model the interaction between two immiscible fluids by considering the variation of some fluids’ properties across the interface such as surface tension, contact angle and wet boundaries within the microfluidic channels. This method introduces an additional scalar field (the level set function) to the modeling domain where the transition between fields vary from 0 to 1, thus defining an appropriate resolution between phases (see Supplementary Materials, File S1, Code S1, Video S1, and Video S2 for further information about details on the description of the level set function).

The governing equations for the level set method are a version of the convection-diffusion equation, with the advective term coming from the Navier-Stokes equations (Equation (1)).

(1) $$\frac{\partial \Phi }{\partial t}+u\nabla \Phi =\gamma \nabla \left(\epsilon \nabla \Phi -\Phi \left(1-\Phi \right)\frac{\nabla \Phi }{\left|\nabla \Phi \right|}\right)$$

Equation (1) is a version of the advection-diffusion equation implemented during Multiphysics simulations. The level set method is used to model moving interfaces such as the two immiscible phases used in the experimental process to form the capsules. The main advantage of this approach is the ability to represent moving interfaces using a fixed mesh. As a result, the modeling interface solves Equation (1) in order to move the interface with a velocity field *u.* The interface is represented by a level set of a globally defined function, function (*Φ*), which consists of a step function that equals zero in one domain and one in the other, hence at the interface there is a smooth transition from zero to one. The interface is defined by the 0.5 isocontour, or the level-set, of *Φ*.

The terms on the left-hand side of Equation (1) define the correct motion of the interface, as it corresponds to the convection term, while those on the right-hand side are established for numerical stability and correspond to a version of the diffusion term of the generalized convection-diffusion equation. The parameter ϵ determines the thickness of the region where *Φ* goes from zero to one, and it is generally of the same order as the size of the elements of the mesh. This parameter is constant within each domain. The parameter $\gamma \;\left[\frac{N}{m^{2}}\right]$ is related to the amount of reinitialization or stabilization of the level set function. Small values of γ might lead to oscillations in the level set function because under such circumstances the thickness of the interface fails to remain constant. In contrast, large values of γ lead to incorrect movement of the interface [43]. A suitable value for stable and meaningful simulations was the maximum magnitude of the velocity field $\left[\frac{m}{s}\right]$. Since this problem deals with a conservative approach, the volume (area for 2D problem) bounded by the interface should be conserved in the absence of inflows or outflows through the boundaries, ∇u=0.

By default, the level set method uses a non-conservative approach where only the integral of the level set function is conserved. This approach has proven to be better suited for numerical calculations and provides rapid convergence.

The imposed boundary conditions were the no-slip condition at the channels’ walls, $V_{h}=0$ mm/s, and the pressure at the outlet, atmospheric pressure ($P_{o}=1\;\mathrm{atm}$), to suppress backflow (see Figure 1B). The velocities of the continuous phase and discrete phases were set to 4.98 mL/h and 169.98 mL/h, respectively. These values were estimated by dividing experimental flow rates by the channel cross section area at the droplet generation junction.

To assure relatively short computation, a 2D geometry of the device was implemented in COMSOL (Figure 1B). Water (liquid) and vegetable oil were selected as dispersed and continuous phases from the materials library of COMSOL. This allowed us to retrieve the required properties for the simulations including interfacial tension, density and dynamic viscosity. To evaluate droplet generation, a two-step phase initialization and time dependent study was executed over 6 seconds with 0.01 s-time steps. A parametric sweep was conducted to estimate changes in the microcapsule size distribution as a function of operation conditions. Accordingly, the flow rate of the continuous phase was set to 83, 125, 167, 209, 251 and 293 mL/h, while in the case of the dispersed phase, the values were 1, 2, 3 and 5 mL/h. Additionally, three concentrations of sodium alginate were studied, namely, 0.5%, 0.7% and 1.0% (w/v). The simulation results were directly compared with those obtained experimentally.

### 2.3. Fabrication of the Microfluidic Device for Microcapsules Generation

**Figure 2:**
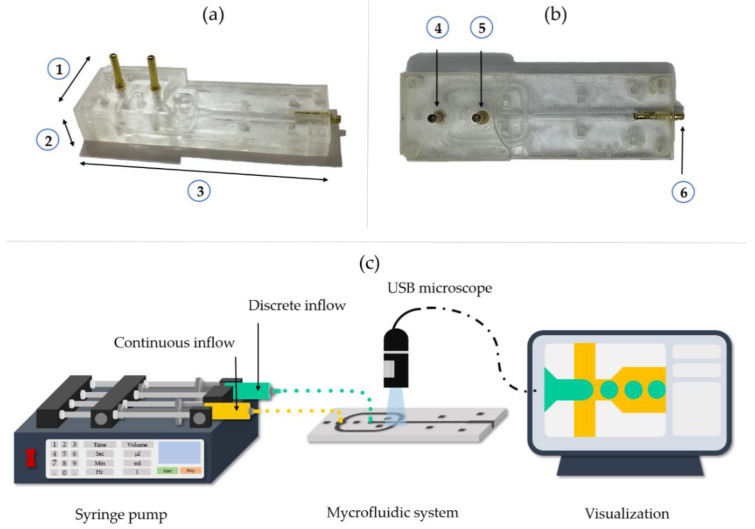
Final microfluidic device assembled with (**a**) dimension of ① 251 mm width, ② 127 mm height and ③ 757 mm length. (**b**) Top view of device with ④ continuous phase inlet, ⑤ dispersed phase inlet, and ⑥ flow outlet. (**c**) Schematic of the setup for microcapsules manufacture and video collection and analysis.

Laser cutting (TROTEC ^®^ Speedy 100, 60 w laser cutter, TROTEC, Marchtrenk, Austria) was used as a physical technique for the fabrication of the prototype onto the PMMA substrate. Device parts were manually aligned and sealed layer by layer with the solvent adhesive methylene chloride, while maintaining a constant pressure on the system using a homemade press. Proper sealing was tested by pumping water through the system. Syringes of 5 mL and 50 mL were connected at the inlets of the system, while water was pumped with the aid of syringe pumps (78-8110C Programmable Touch Screen, Cole-Parmer^®^, Vernon Hills, IL, USA and B Braun Perfusor^®^ compact, B. Braun, Melsungen, Germany). The dimensions of the final assembled device (Figure 2a,b) were 251 mm width, 127 mm height and 757 mm length. The final device consisted of 5 layers of PMMA, where three of them helped in assembling the microchannel section of the device, while the remaining two facilitated the introduction of the solutions to the systems.

### 2.4. Fabrication and Characterization of Microcapsules

Sodium alginate solution 1% (w/v) was prepared by dissolving 0.5 g of sodium alginate in 50 mL of milli-Q water at 800 rpm for 2 h until a homogeneous solution was obtained. Microcapsules were fabricated with the aid of the manufactured device by infusing the solution of sodium alginate with a 78-8110C Programmable Touch Screen syringe pump (Cole-Parmer^®^, Vernon Hills, IL, USA). Likewise, commercial soybean vegetable oil was injected as continuous phase with a B Braun Perfusor^®^ compact syringe pump (Figure 2c). The resulting mixture with the alginate solution drops was immediately added to a 3% (w/v) CuSO_4_ solution for microcapsule gelation at room temperature for 30 min. The resulting particles were subsequently filtered out with grade 4 qualitative filter paper. The system was evaluated under the same operation conditions studied in silico, i.e., three concentrations of sodium alginate (0.5, 0.7 and 1% (w/v)), six different flow rates in the Q_c_ and four flow rates for the Q_d_.

The microsystem under operation was recorded with a 1000× Digital USB Microscope. The size distribution of the microcapsules was estimated from the recorded videos using a homemade application developed in MathWorks MATLAB^®^ (MathWorks, Natick, MA, USA). The implemented algorithm was based on Hough transform to detect the microcapsules as circles after processing frames and binarizing each image. The resulting circles were initially measured in pixels followed by a transformation into distance using a previously established correlation between µm and pixels in the frame.

### 2.5. Enzyme Encapsulation

Initial laccase solution was obtained by dissolving an amount of enzyme preparation in milli-Q water to reach 2800 U/L. 1 mL of laccase solution (2800 U/L) was mixed with 4 mL of 1% (w/v) sodium alginate solution. The obtained solution was then employed as the Q_d_ fluid, while commercial vegetable oil was employed as the Q_c_ fluid. The flow rates for the microcapsule generation (169.98 mL/h for the continuous flow and 4.98 mL/h for the discrete flow) were established using a B Braun Perfusor^®^ compact syringe pump and a 78-8110C Programmable Touch Screen (Cole-Parmer^®^, Vernon Hills, IL, USA) syringe pump equipped with syringes of 50 mL and 10 mL, respectively.

Before microcapsule generation, the system was purged with vegetable oil and the exit of the system was connected to a 3% (w/v) copper sulphate solution. Once the capsules were formed inside the device, they were cross-linked in the copper sulphate solution for about 8 h. Finally, the capsules were filtered out using a Buchner funnel and stored at room temperature until further use.

### 2.6. Free and Microencapsulated Laccase Activity

Free enzyme activity measurements were performed as reported by Niku-Paavola et al. [44]. Phosphate buffer was prepared from KH_2_PO_4_ and adjusted to the required pH with 1M NaOH or 1M HCl solutions. The activity of the sample was measured in a Genesis 10S spectrophotometer (Thermo Fisher Scientific, Waltham, MA, USA) for 1 min at 436 nm. One activity unit was defined as the amount of laccase needed to oxidize 1 µmol of ABTS per minute under the assay conditions. Laccase activity was expressed in terms of units per liter (U/L).

Encapsulated enzyme activity was determined using an indirect measurement as the difference between free laccase activity and residual enzyme activity on the supernatant after filtration. This is because according to a mass balance, the total enzyme units can be approximated by the sum of those present in the encapsulated system and the ones remaining in the supernatant after encapsulation. As a result, the immobilization ratio and activity recovery can be calculated according to Equation (2). Where *A* refers to enzyme activity in the initial solution before encapsulation and *B* the activity of laccase in the supernatant solution of copper sulfate after immobilization. Measurements were carried out in triplicate.

(2) $$Immobilization\;ratio\;\left(\%\right)=\frac{A-B}{A}*100\;$$

Activity of the encapsulated enzyme was also assessed by direct visualization under an optical microscope at 40× magnification. Microcapsules were exposed to 200 µL of ABTS solution (11 mg/mL) in milli-Q water, and the color changes due to biotransformation of the ABTS were observed for 5 min.

### 2.7. Encapsulated Laccase Characterization

Laccase was labeled with Rhodamine B (RhB) for confocal microscopy visualization. Fluorophore conjugation was achieved in a 40% (v/v) DMF medium with EDC and NHS as coupling agents to form amide bonds between the carboxyl groups of RhB and the free amines of the laccase molecules. Purification of the labeled enzyme was via ultracentrifugation with a cellulose membrane of MWCO 10 KDa (Merck, Darmstadt, Germany).

Microcapsules were then fabricated following the same procedure described above. Laser scanning confocal microscope fluorescence images of microcapsules with labeled enzyme were obtained using a confocal microscope FLUOVIEW FV1000 (Olympus, Tokyo, Japan). Samples were examined with a 559 nm laser as light source for RhB excitation at 10× magnification and 0.4 (NA). The potency of the laser was 37% with a transmitted light system and an Alexa Fluor 568 filter. The laccase microspheres were prepared and observed on a Jeol Lyra 3 (TESCAN, Brno, Czech Republic) Focused Ion Beam-Scanning Electron Microscope (FIB-SEM, TESCAN instrument, Brno, Czech Republic). The observation was carried out in a cooling stage system. A beam potency of 10 kV was used at 80× and 1500× magnification.

## 3. Results

### 3.1. Multiphysics Simulations

The Multiphysics simulation allowed us to visualize the rate of droplet generation by plotting the time evolution of volume fraction changes and the velocity profile within the channels. Fluid velocities were calculated by dividing flow rates values by the channel cross-section area at the junction section of the microfluidic system.

**Figure 3:**
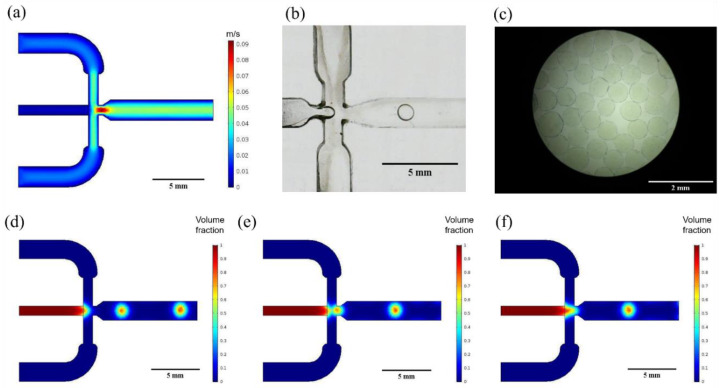
Simulation and experimental results. (**a**) Velocity profile for an average dispersed phase velocity of V_d_ = 15.73 mm/s and an average continuous phase velocity of V_c_ = 4.56 mm/s and (**b**) experimental droplet formation with the assembled microfluidic system for Q_d_ = 4.98 mL/h and Q_c_ = 169.98 mL/h. Dispersed phase volume fraction to evaluate droplet generation at microfluidic junction. (**c**) Optical microscope images of produced microcapsules magnification 40×, (**d**) dispersed phase deformation, (**e**) droplet pinch-off, (**f**) final droplet formation.

Figure 3a shows the velocity profile as recovered from COMSOL Multiphysics simulations. The initial section of the three inlets presents a constant velocity with a further acceleration while approaching the droplet generation junction. Maximum fluid velocity is then achieved over the generation nozzle due to the cross section narrowing, and then it eventually decelerates at the outlet section due to the expansion of the flow cross sectional area.

Figure 3d–f show the different droplet generation stages according to the results of the multiphase flow level-set simulations. The generation profile corresponds to a flow focusing microfluidic geometry where continuous phase fluid flows on either side of the dispersed phase fluid to an orifice in the microfluidic device nozzle. As described by Nunes et al. [45], the elongational velocity field of the continuous phase fluid stretches the dispersed phase to a thin jet, which eventually breaks into droplets.

**Figure A1:**
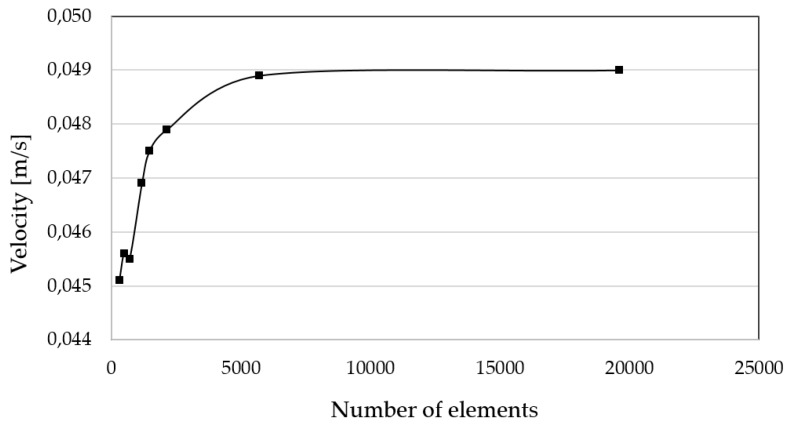
Mesh convergence plot. The convergence of above 6000 elements was assured as demonstrated by changes below 3% in the average velocity of the fluid.

The mesh convergence study shows that above ~6000 mesh elements, the change in the magnitude of the velocity is less than 3% (Appendix A, Figure A1).

### 3.2. Microcapsules Size Analysis

**Figure 4:**
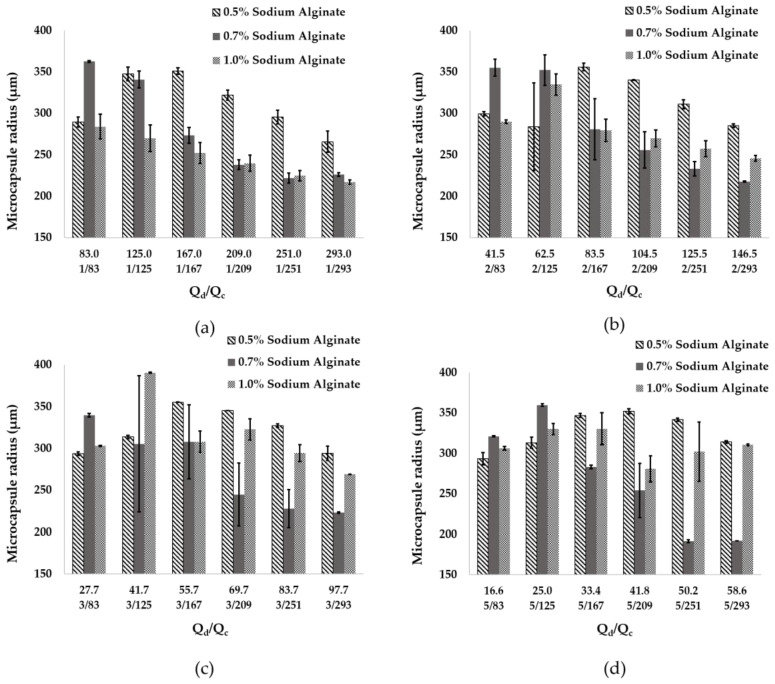
Mean radius of microcapsules found for alginate concentrations of 0.5%, 0.7% and 1% (w/v) at Q_c_ values of 83, 125, 167, 209, 251 and 293 mL/h, for Q_d_ of (**a**) 1 mL/h, (**b**) 2 mL/h, (**c**) 3 mL/h and (**d**) 5 mL/h.

According to recent reports, for flow focusing microfluidic systems, variations of input flow rates, concentration of the reagents and the channel diameter might have a significant impact on microcapsule size distribution [46,47]. For this reason, here we explored the combination of six different Q_c_ values and five different Q_d_ values. The microcapsule mean size was analyzed for 30 s for each flow ratio (Q_d_/Q_c_) using the developed MATLAB^®^ application. Figure 4 shows the microcapsule radius as a function of the Q_d_/Q_c_ ratio.

For alginate concentrations of 0.5%, 0.7% and 1% (w/v), the mean radio of microcapsules found of the 24 flow ratios studied for each concentration were 318.2 ± 6.4 µm, 275.3 ± 15.0 µm and 288.0 ± 9.7 µm, respectively. The smallest radius of alginate microcapsules of about 191.6 ± 1.8 µm was found using a concentration of 0.7% (w/v) of alginate and flow rates of 251 mL/h (Q_c_) and 5 mL/h (Q_d_). The largest capsule size was 390.5 ± 1.7 µm for a Q_d_/Q_c_ ratio of 3/125. A further increase of Q_c_ to 293 mL/h was attempted to reduce capsule size with no success. This was attributed to the relatively large channel diameters of about 1 mm. We hypothesize that microcapsule size could be potentially reduced by changing the Q_d_/Q_c_ ratios in a device with smaller channel diameters [47].

**Figure 5:**
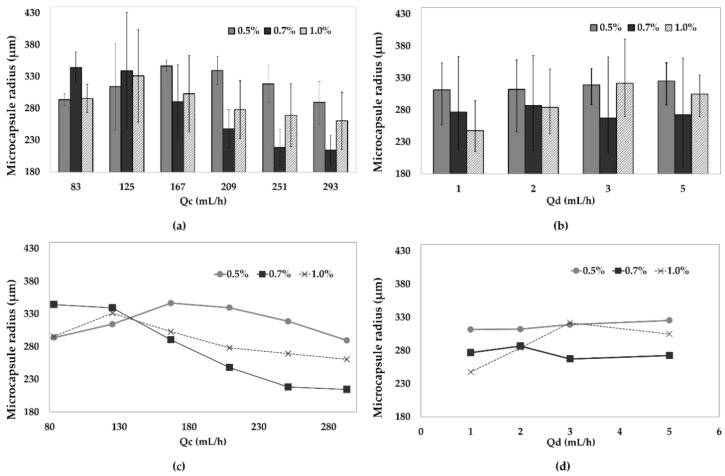
Mean radius of microcapsules found for alginate concentrations of 0.5%, 0.7% and 1% (w/v) (**a**,**c**) modifying only Q_c_ rate, and (**b**,**d**) modifying only Q_d_ rate. Plots (**c**,**d**) are simplified plots of (**a**,**b**), respectively, where only the mean radius was included.

The size of the capsules decreased with an increase in the Q_c_ flow above 130 mL/h for all alginate concentrations with the exception of 0.5% and 1.0% (w/v) for Q_d_ rates higher than 3 mL/h. However, overall changes in Q_d_ flow rate showed no significant impact on the capsule size for alginate concentrations below 1.0% (w/v) (Figure 5).

**Figure 6:**
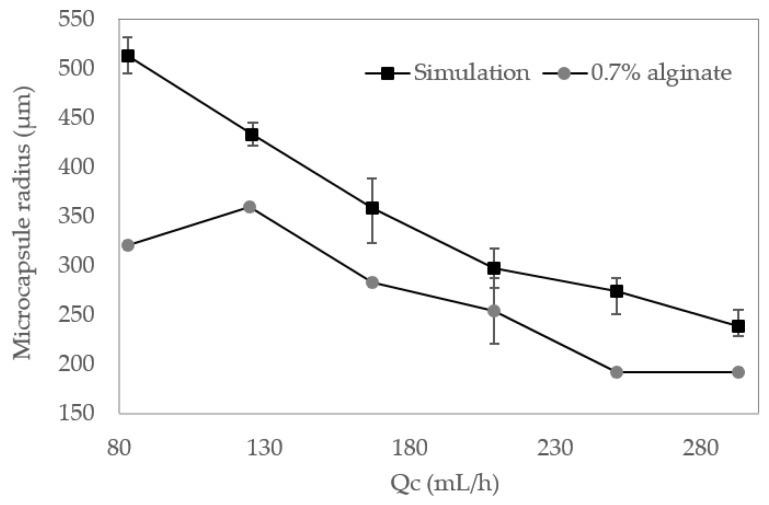
Mean radius of microcapsules found during Multiphysics simulations and experimental results at 0.7% (w/v) alginate, Q_d_ = 5 mL/h and Q_c_ varied from 83 to 293 mL/h.

The size distribution was also calculated for the particles generated in silico via Multiphysics simulations. Particle diameters were analyzed using the open-source software ImageJ^®^ for a dispersed phase flow rate (Q_d_) of 5 mL/h. The continuous phase flow rate (Q_c_) was varied from 83 to 293 mL/h (Figure 6). It was found that the particle size is inversely proportional to the (Q_d_/Q_c_) ratio, which is in line with the model proposed by Nunes et al. [45] for a dripping regime. Accordingly, a maximum radius of 512 ± 19 µm was estimated for a Q_d_/Q_c_ of 5/83, which decreased to 238.25 ± 16.75 µm for a Q_d_/Q_c_ of 5/293. Even though experimental results showed a mean size 100 µm smaller than simulation results, at a 0.7% (w/v) alginate concentration, sizes obtained from the two methods have a higher degree of coincidence (Figure 6). These results provide further evidence for the notion that Q_d_ has no significant impact on size, while Q_c_ acts as a dominant parameter for the given experimental set up. By reducing the Qd/Qc ratio while maintaining Qd constant, it is possible to increase the velocity field u and, consequently, the bulk motion of the fluid. As a result, in the left-hand side of Equation (1), the term that contains u and is responsible for modeling mass transfer through the level set function Φ increases as well [43].

### 3.3. Visualization of Enzyme Encapsulation

**Figure 7:**
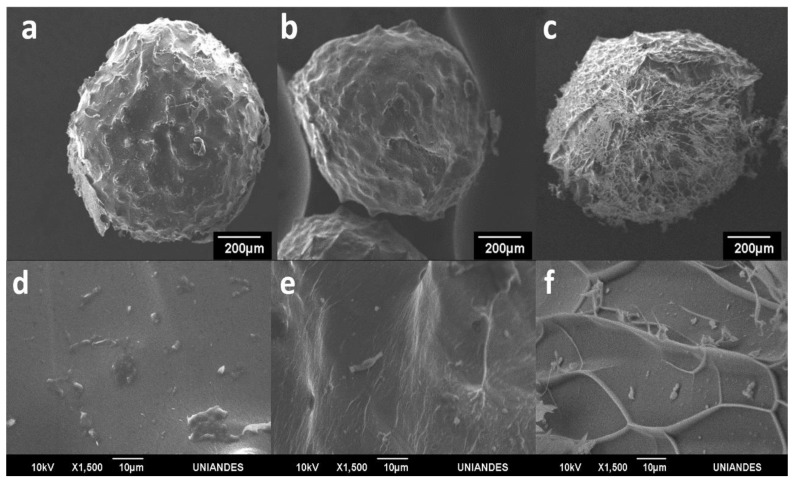
SEM images of laccase-encapsulated microspheres (**a–c**) after 1, 20 and 106 days of fabrication, magnification of 80×; and (**d**–**f**) magnification of 1500×, respectively.

The laccase-encapsulated microspheres observed using a FIB-SEM (Figure 7) exhibited a typical rounded-shape morphology and some surface protrusions; however, as time passed, the surface morphology changed to one that is more fiber-like due to the relaxation and reorganization of the microcapsule’s structures. The stability of ionically cross-linked microcapsules depends on pH, kinetics and thermodynamics. Over time, an important disruption of bonds connecting the alginate monomers is promoted when the material is exposed to different alkaline and acidic environments. Accordingly, capsules have thought to lose the characteristic mechanical properties due to the outflux of cross-linking ions into the medium [48,49,50].

Due to the diameter of the microsystem channel and the flow rates used, a size distribution was obtained in the range of 203.13–716.00 μm, which was not only able to provide a large specific surface area for enzyme immobilization but also make the microspheres easy to separate and recover [51]. Moreover, the microspheres could be dispersed well in aqueous media. These properties are ideal for enzymatic transformations as the substrates and products can be freely exchanged during operation and therefore prevent significant mass transfer limitations. Moreover, from a productivity viewpoint, the material offers the possibility for a large number of enzyme molecules immobilized per unit mass, which could be potentially reflected in reduced costs per mass of transformed substrates.

**Figure 8:**
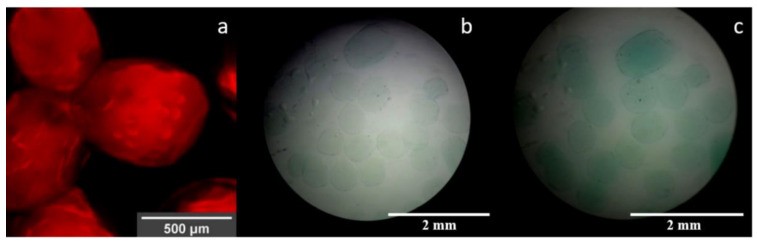
(**a**) Confocal image of laccase labeled with Rhodamine B inside microspheres using magnification of 10×. Optical microscopy images at (**b**) t = 0 min and (**c**) t = 5 min of encapsulated laccase exposed to ABTS solution at 40× magnification.

Successful immobilization of laccase molecules was demonstrated using direct observation of the Rhodamine B-labeled molecules inside the alginate capsules (Figure 8a). The confocal images not only confirmed encapsulation but also their relatively homogeneous surface distribution throughout the material. Even though there is evidence that modified versions of Rhodamine B can be used to assess the presence of copper, here we only considered this fluorophore to qualitatively assess the distribution of the enzyme molecules within the microcapsules [52,53,54]. This was achieved by covalent conjugation of Rhodamine B to the enzyme molecules prior to encapsulation. According to the images presented in Figure 8a, the morphology of microcapsules was not significantly altered by the presence of the labeled molecules. Further evidence of the presence of functional laccase molecules after encapsulation was provided by the ability of the obtained encapsulates to catalyze ABTS (Figure 8b,c).

**Figure 9:**
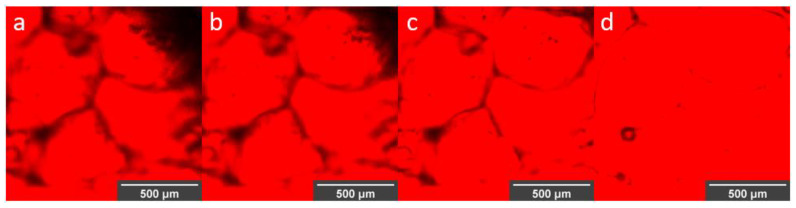
Confocal images of laccase labeled with Rhodamine B migrating out of the alginate microspheres. Time 0 s (**a**), 35 s (**b**), 157 s (**c**) and 600 s (**d**) after fabrication. Magnification of 10×.

Figure 9 shows that once encapsulated, the laccase molecules migrated out of the material most likely by simple diffusion as observed in a time lapse of 0 to 157 s. The labeled enzyme was released out of the capsule, thereby filling the spaces between them (Figure 9d). However, an amount of laccase remained inside the capsules, suggesting that diffusion is a continuous phenomenon that lasts for more than the 10 min plotted in Figure 9 (data not shown).

## 4. Discussion

The encapsulation of biomolecules such as DNA, multi-enzyme systems and antibodies, among many others, have allowed the development of systems capable of supplying such biomolecules in a controlled manner. This is critical for applications in medicine, agriculture, cosmetics and food, where environmental or physiological conditions might be detrimental for the integrity of the encapsulated biomolecules. More specifically, in the case of encapsulated enzymes, these systems prevent their leakage or their coming into direct contact with the external environment. [55]. Recent reports also confirm that enzyme stability is considerably increased upon encapsulation [56]. In this work the calculated free enzyme activity was 1670 U/L after initially adding 1.67 U for capsule fabrication. The final encapsulated enzyme was therefore 1.10 U, which corresponds to an immobilization ratio of 65.94%. The effectiveness of encapsulation has been reported to be highly dependent on the type of enzyme. For instance, in the case of horseradish peroxidase and butyrylcholinesterase, efficiencies might be over 90%, while for lipases and luciferase, values could be well below 20%. Therefore, in our case, the immobilization efficiency achieved could be considered satisfactory [56,57].

The use of a low-cost microfluidic platform provides a simple approach for the precise manufacture of the capsules with controlled size distribution, shell thickness and permeability, which guarantees good reproducibility and a more predictable release behavior [58,59]. The dimensions, topology and flow parameters of the microfluidic system appear to be important in controlling the resulting size and morphology of the alginate capsules. Simulations allowed us to predict that the selected geometries led to a considerable increase in the continuous phase velocity as the fluid approaches the narrowing section right over the droplet generation nozzle (Figure 3a). This channel reduction and the corresponding pressure drop as the channel widens is responsible for an adequate dripping regime where droplets can be successfully formed. Reaching this regime allowed us to avoid possible dispersed phase flow jets that may ultimately interfere and destabilize droplet generation [45]. Similar to the results of other works [60], the simulations and experimental results concur that microcapsule size decreases with an increase of Q_c_. However, in this work, microcapsule mean sizes obtained in silico were almost 50 µm higher at each Q_d_/Q_c_ ratio compared with those obtained experimentally, and we believe that this result could be explained by the imposed boundary conditions and materials parameters used for the numerical study. The simulations also predicted that in the absence of enzyme the average size of the capsules approached 330.3 ± 19.7 μm, while the ones obtained experimentally exhibited an average of 293.8 ± 10.4 μm.

Enzyme encapsulation proceeded by adding it to the alginate solution prior to entering the microfluidic device. Once the droplets are formed, encapsulation is finalized by cross-linking in the presence of the CuSO_4_ 3% (w/v) solution. These copper atoms may play a role in altering the activity of native laccase molecules as they have been reported to allow the storage of electrons during the oxidation processes. In this regard, addition of CuSO_4_ to fungus cultures resulted in a substantial increase in laccase activity [61]. Notwithstanding, there is also evidence of a possible decrease in enzyme activity due to the presence of copper salts [62]. Here, however, we disregarded possible effects of Cu^+2^ on the activity of encapsulated laccase molecules. The fabricated capsules had an ample size distribution in the range of 203.13–716.00 μm, which can be attributed to the ability of the enzyme molecules to alter physical properties of the alginate solution such as viscosity.

The resulting microcapsules work as semi-permeable containers capable of encapsulating molecules by physically trapping them within the cavities formed when the polymer is cross-linked with the Cu^2+^ ions [63]. The stability and release kinetics of the ionically cross-linked alginate microcapsules are then limited and controlled by their environmental conditions, which can alter the movement of the structural divalent ions into the surrounding media [25]. In different applications, the release of molecules from alginate microcapsules was shown to be controlled by diffusion, the alginate concentration in the cross-linked material and the extracapsular environmental pH [64,65]. Moreover, the degradation of capsules might lead to the release of common cross-linkers such as Ca^2+^, which can be significantly relevant for the case of biomedical applications where the presence of such ions in vivo can lead to altered hemostasis, which can be desirable or not depending on the situation [25,66]. Confocal microscopy showed a fast enzyme release in water starting short after capsule fabrication. Therefore, enzyme release from these microcapsules can be related, first, to the passive diffusion through the pores of the material and, second, to the material decay in response to environmental conditions.

## 5. Conclusions

Capsule size is inversely proportional to the ratio of the flow rates for the continuous and dispersed phase. Multiphysics simulations the viability of the microfluidic system to be assessed for droplet generation and provided critical information regarding capsule generation frequency and size distribution as a function of flow rates. The obtained results were validated experimentally with acceptable agreement. For instance, a discrepancy of only 50 μm was observed for particle size. The value of Q_c_ was found to play a significant role in controlling the particle size of the capsules for the given experimental set up. Spectroscopic and microscopic characterization confirmed effective enzyme encapsulation of 65.94% of the initial laccase.

As low-cost materials and inexpensive manufacture processes were employed for assembling the microfluidic device for the fabrication of capsules, this approach represents a convenient avenue for producing laccase encapsulates in relatively large amounts with a controlled size distribution and a superior encapsulation capacity.

## Supplementary Materials

The following are available online at https://www.mdpi.com/2073-4360/12/5/1158/s1, File S1: Supplementary material description, Code S1: convection_diffusion_eq.m, Video S1: Level_Set_u_05.avi, Video S2: Level_Set_u_15.avi.

## Appendix A. Mesh Convergence Study

Figure A1 shows the mesh convergence study results. Above ~6000 mesh elements, the change in the magnitude of the velocity is less than 3%.
