# Supplementary material for: Fabrication and Characterization of a Low-Cost Microfluidic System for the Manufacture of Alginate–Lacasse Microcapsules
Source: Polymers (Basel). 2020 May 19;12(5):1158. doi: 10.3390/polym12051158 (PMC7284885; doi:10.3390/polym12051158)
Supplement: Supplementary file 1 [file polymers-12-01158-s001.zip › polymers-774058-supplmentary-final/Supplementary material/polymers-774058-supplementary materials.docx]

Supplementary Material

Fabrication and Characterization of a Low-Cost Microfluidic System for the Manufacture of Alginate–Lacasse Microcapsules

**MATLAB code: Simulation**

The *convection_diffusion_eq.m* code simulates the behavior of a level set function, which is used in the level-set Multiphysics in COMSOL in order to model the interaction between two immiscible species. This model describes the evolution of the function over time and modeling domain.

Initially we define our variables which are simulation time, timestep, number of nodes (N) and velocity v which represents the velocity field. As for visual representation and better comprehension the function is first described as a gaussian function.

Following, an upwind scheme is set as numerical discretization method which is used to solve hyperbolic partial differential equations by using differencing in the direction determined by the sign of the given speed.

To illustrate this method, consider one dimensional linear advection given by Equation (S1)

| $\frac{\partial\Phi}{\partial t}+u\frac{\partial\Phi}{\partial x}=0$ | (S1) |
| --- | --- |
| $\frac{\partial\Phi}{\partial t}+u\nabla\Phi=\gamma\nabla\left( \epsilon\nabla\Phi-\Phi(1-\Phi)\frac{\nabla\Phi}{\left\vert\nabla\Phi\right\vert} \right)$ | (S2) |

Which models a first-order wave propagation over the x-axis, where its direction depends on the sign of parameter u, since u > 0, the waves moves to the right side of the axis being a upwind scheme. By adding right hand side in Equation (S2) we consider the diffusion term used in the generalized convection diffusion equation, in function of the given level-set. As a boundary condition the interface between the two phases is specified by $\Phi\left( x,t \right)=0.5$.

**Supporting videos**

Supporting videos illustrate the behavior of the advection-diffusion equation implemented during simulations

These allowed to verify that by increasing the velocity field parameter, the rate of phase transition is reached more rapidly, surpassing the $\Phi\left( x,t \right)=0.5$ limit. Therefore, the mass transition that models the function is increased due to the bulk motion of the fluid.
